# Small elements, huge impact: the role of micronutrients in pemphigus

**DOI:** 10.3389/fimmu.2026.1980495

**Published:** 2026-10-08

**Authors:** Hong-mei Chang, Xue Wang, Juan Du, Wei-long Zhang

**Affiliations:** 1 Department of Oral and Maxillofacial Surgery, Shandong Provincial Hospital Affiliated to Shandong First Medical University, Jinan, Shandong, China; 2 School of Stomatology, Shandong First Medical University, Jinan, China

**Keywords:** antioxidants, micronutrient, pemphigus, trace elements, vitamins

## Abstract

Pemphigus is an autoimmune blistering disease affecting both the skin and mucous membranes, causing secondary infections and high mortality. Micronutrients are important factors influencing the health of oral mucosa. It has been reported that patients with pemphigus have reduced levels of vitamins A, B3, D, copper, iron, selenium, and antioxidants. However, the specific mechanistic role of micronutrients in the initiation and progression of pemphigus remains poorly characterized. This review presents a detailed exploration of the relationship between pemphigus and micronutrient levels, seeking to unravel the mechanistic basis of micronutrients’ effects on pemphigus pathogenesis and to discuss their prospective applications in the disease’s prevention and therapy.

## 1 Introduction

Pemphigus encompasses a set of distinct autoimmune bullous diseases affecting the skin, mucous membranes, and skin appendages (1). The classification of its subtypes is predominantly based on the specific desmosomal or non-desmosomal antigens targeted by pathogenic autoantibodies (2). Clinically, pemphigus can be categorized into five major subtypes, namely pemphigus vulgaris (PV), pemphigus foliaceus (PF), pemphigus herpetiformis, paraneoplastic pemphigus (PNP), and IgA pemphigus.

The prevalence of pemphigus exhibits distinct regional and ethnic disparities. As reported by Kridin et al., individuals of Ashkenazi Jewish or Mediterranean ancestry have a markedly higher prevalence of this disease (1, 3). Moreover, the incidence of different pemphigus subtypes varies considerably across geographic regions. Among them, PV and PF are the two most common types: PV makes up about 70% of all pemphigus cases, while PF accounts for approximately 20%–30% (2). Additionally, pemphigus is linked to notable morbidity and mortality. The severe blisters caused by the disease may lead to fatal consequences due to secondary infections and malnutrition, with the annual mortality rate being 5%–15% (4). What is more, the changes in appearance (such as flaccid blisters and depigmentation of skin and mucosa) caused by the disease, together with the side effects of long-term use of glucocorticoids, will affect patients’ self-esteem and thus bring about psychological trauma (4).

Pemphigus is pathologically defined by the loss of intercellular adhesion among epidermal keratinocytes, driven by pathogenic autoantibodies targeting desmoglein 1 (anti-Dsg1) or desmoglein 3 (anti-Dsg3). This antibody-mediated disruption induces acantholysis and intraepidermal blister formation, which clinically present as flaccid blisters and erosions on the skin and/or mucous membranes (5, 6). The profile of patchogenic anti-desmoglein antibodies exhibits distinct subtype-specificity in pemphigus (7). Dsg1 is widely expressed across all layers of the cutaneous epidermis, whereas Dsg3is predominantly localized to the basal and immediate suprabasal layers of the epidermis and more abundantly expressed in mucosal epithelia. In PF, the immune system specifically produces anti-Dsg1 antibodies. These antibodies cause superficial blisters and skin erosions, with no mucosal involvement — this aligns with Dsg1’ s distribution in the epidermis. Conversely, PV is characterized by the production of anti-Dsg3 antibodies, either alone or in combination with anti-Dsg1. These antibodies disrupt intercellular adhesion in the deeper epidermal layers, resulting in severe and often refractory mucosal erosions; the concurrent presence of anti-Dsg1 typically correlates with additional cutaneous involvement (3, 8).

The etiology of pemphigus remains incompletely elucidated. Accumulating evidence indicates that multiple etiological factors, including medications, infections, vaccines, radiation exposure, psychological stress, and dietary components, play pivotal roles in driving the pathogenesis of this disease. In particular, as an important part of the diet, micronutrients have been recognized as potential triggers for the pemphigus (9). Micronutrients include vitamins g(e.g., vitamins A, B, and D), trace elements (e.g., copper, iron, and selenium), and antioxidants. A sufficient intake of micronutrients is essential for the optimal function of the immune system (10), whereas imbalances in these nutrients may contribute to the onset and progression of various disorders (11). For instance, vitamin D exerts immunomodulatory effects via the vitamin D receptor (VDR) pathway: it promotes the secretion of anti-inflammatory cytokines such as interleukin (IL)-10, directly inhibits pathogenic T helper 17 (Th17) cells and their key pro-inflammatory effector cytokine IL-17, and suppresses autoantibody-producing B lymphocytes. This process regulates immune dysfunction in pemphigus, reduces Dsg3 production, and thus eases the loosening of epidermal cells in the stratum spinosum. Consequently, vitamin D supplementation may be helpful to alleviate clinical symptoms of pemphigus (12).

Nevertheless, the mechanistic link between dynamic changes in micronutrient concentrations and the progression of pemphigus remains incompletely understood. This review endeavors to elaborate on the specific contributions of essential trace elements, vitamins, and antioxidants to the immunopathological processes of pemphigus, with the ultimate goal of paving the way for precise prevention and targeted therapy of the disease.

## 2 Methods

### 2.1 Protocol

This systematic review was conducted in accordance with the Preferred Reporting Items for Systematic Reviews and Meta-Analyses (PRISMA) 2020 guidelines to ensure transparent and complete reporting (13). The review protocol was not prospectively registered in an open registry such as PROSPERO. Ethical approval was not required because this study did not involve primary data collection, patient recruitment, or any clinical intervention. The review question, eligibility criteria, and analytical approaches were predefined using the PICOS (Population, Intervention/Exposure, Comparison, Outcome, Study design) framework and consistently applied throughout the review process.

### 2.2 Eligibility criteria

Study eligibility was defined based on the PICOS framework. Population: Patients diagnosed with pemphigus (including all subtypes, clinically and histopathologically confirmed based on modified WHO or Krutchkoff-Eisenberg criteria.

Intervention/Exposure: Measurement of micronutrient levels (including vitamins, antioxidants, and trace elements) in serum, plasma, saliva, tissue; or intervention with micronutrient supplementation. Comparison: Healthy controls without pemphigus, or disease severity subgroups where healthy controls were unavailable. Outcome: Prevalence and levels of micronutrient deficiencies in pemphigus patients. Study design: Original articles including case-control studies, cross-sectional study, randomized controlled double-blind trial, and *in vitro* cell studies.

Inclusion criteria: original articles including case-control, cross-sectional, cohort studies, and clinical trials; assessment of at least one micronutrient in pemphigus patients; confirmation of pemphigus clinically and histopathologically based on modified WHO or Krutchkoff-Eisenberg criteria; measurement of at least one micronutrient level in serum, plasma, saliva, tissue, or exudate; interventional studies evaluating the efficacy of micronutrients in the management of pemphigus also assessed lesion response; and articles published in English.

Exclusion criteria: case reports, case series, reviews, meta-analyses, abstracts, commentaries, letters to the editor, opinion articles, animal studies, evaluation of toxic elements, other oral disorders, pemphigoid instead of pemphigus, studies diagnosing pemphigus according to no reliable criteria or showing evidence of tissue dysplasia, and non-comparative studies. For studies (e.g., Marzano et al.) that enrolled both PV and BP, only data specific to PV were extracted and included in the synthesis.

### 2.3 Information sources and search strategy

Three electronic databases including PubMed, ScienceDirect, and Web of Science were searched from database inception to March 2026 without additional time limit restrictions. The core search terms were consistent across databases, with minor syntax adjustments to adapt to platform specific retrieval rules.

The primary search syntax was as follows: pemphigus AND (trace element OR micronutrient OR vitamin A OR retinoid OR vitamin B OR nicotinamide OR vitamin D OR iron OR copper OR selenium OR antioxidant OR oxidant).

All retrieved records from three databases were imported into EndNote for subsequent deduplication and screening.

### 2.4 Study selection

The study selection process was conducted by two independent authors (H-M.C. and X.W) in accordance with the PRISMA 2020 guidelines. The disagreement was resolved by corresponding authors. Initially, all records retrieved from the database searches were imported into EndNote (Clarivate Analytics, Philadelphia, PA, USA) for reference management. Duplicate records were removed first. Remaining articles were screened by title and abstract against the pre-specified eligibility criteria; obviously irrelevant records (e.g., reviews, animal studies, papers unrelated to pemphigus or micronutrients) were excluded at this stage.

Full text manuscripts of potentially eligible publications were retrieved and evaluated for final inclusion. Reasons for exclusion of full text articles were documented. The complete selection workflow (identification, screening, eligibility assessment, final inclusion) was visualized in a PRISMA 2020 flow diagram (**Figure 1**). No automated screening tools were used; all inclusion exclusion decisions were made manually by the authors.

**Figure 1:**
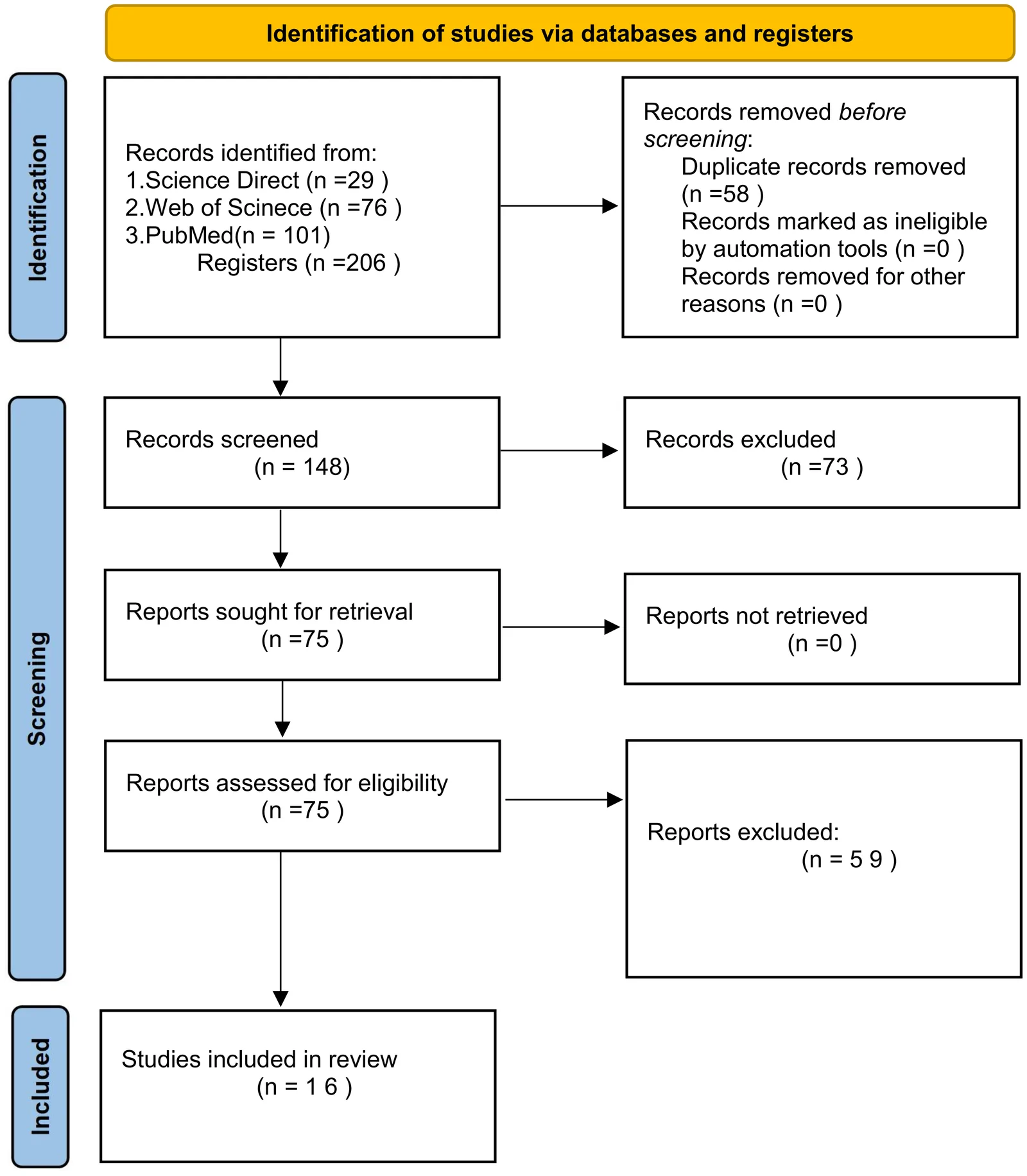
PRISMA 2020 flow diagram showing identification, screening, eligibility evaluation, and inclusion of studies eligible for the systematic review on micronutrients in pemphigus. Figure created using biorender.com.

### 2.5 Data extraction

A standardized Excel spreadsheet was used for data extraction by authors (H-M.C. and X.W). The extracted information including study ID, type of micronutrient, study design, study groups, results, *p* value and the Newcastle-Ottawa Scale (NOS), as presented in **Table 1**.

**Table 1:** List of articles that evaluated serum levels of micronutrients in pemphigus patients.

Reference	Micronutrient	Study design	Study groups	Results	*p* value	NOS
M.H. El-Komy et al. (14)	Vit D	Case-control	PV:40 Controls:40	PV<Controls	*p* < 0.001	8
M. Zarei et al. (15)	Vit D	Case-control	PV:32 Controls:36	PV<Controls	*p*<0.009	8
A.V. Marzano et al. (16)	Vit D	Case-control	PV:13 Controls:13	PV<Controls	*p* = 0.012	7
A.V. Marzano et al. (17)	Vit D	Case-control	PV:35 Controls:35	PV<Controls	*p* < 0.001	7
H. Moravvej et al. (18)	Vit D	Case-control	PV:52 Controls:56	PV<Controls	*p =* 0.13	7
N. Joshi et al. (19)	Vit D	Case-control	PV:30 Controls:10	PV<Controls	*p=* 0.6958	7
M. Banihashemi et al. (20)	Vit D	Case-control	PV:20 Controls:20	PV<Controls	*p ≤ 0.05*	7
M. Javanbakht et al. (21)	Cu	Case-control	Pemphigus: 43 Controls: 58	The correlations are not significant.	*p* > 0.05	7
H.M. Mashaly et al. (22)	Cu, Zn	Case-control	PV:25 Controls:25	Cu: PV < Controls Zn: PV < Controls	Cu: *p* < 0.016 Zn: *p* < 0.003	5
L. La Serra et al. (23)	Pb, Se	Case‐control	Pemphigus:118 Controls:130	Pb: PV>Controls Se: PV, PF<Controls	Pb: *p* = 0.05 Se: *p* = 0.9	6
M.J. Yazdanpanah et al. (24)	Cu	Case-control	PV:25 Controls:25	Cu: Controls<PV	Cu: *p* < 0.01	7
Y. Yesilova et al. (25)	OSI	Case-control	PV: 27 Controls:24	PV>Controls	*p* = 0.001	5
M. Naziroğlu et al. (26)	Vit A, Vit E, GSH	Case-control	PV: 18 Controls: 18	Vit A: PV<Controls Vit E: PV<Controls GSH: PV<Controls	Vit A: *p*<0.001 Vit E: p<0.001 GSH: p<0.05	6
O. Abida et al. (27)	MDA, CAT, SH	Case-control	PV:36 PF:42 Controls:78	MDA: PV\PF>Controls CAT: PV\PF>Controls SH: no significant difference	MDA: *p* < 0.001 CAT: *p* < 0.001 SH: *p*>0.05	6
JA.A. Shah et al. (28)	TAC	Case-control	PV: 47 Controls:25	PV<Controls	*p* < 0.001	6
N.A. Abdelkader et al. (29)	CoQ10	Case-control	PV: 20 Controls: 20	PV<Controls	*p* = 0.001	5

All *p* values considered as significant if they were less than 0.05.

PV, PV patients; PF, PF patients; NOS, Newcastle-Ottawa Scale; RCDT, Randomized Controlled Double-blind Trial; Vit A, Vitamin A; Vit B, Vitamin B; Vit D, Vitamin D; Vit D3, Vitamin D3; Hcy, Homocysteine, Fe, Iron; Mg, Magnesium; Ca, Calcium; Cu, Copper; Pb, Lead; Se, Selenium; Hcy, Homocysteine; OS, Oxidative stress; GSH, glutathione; MDA, Malondialdehyde; CAT, Catalase; SH, thiol groups**;** TAC, Total Antioxidant Capacity; CoQ10Coenzyme Q10

Micronutrient levels were measured across heterogeneous sample types (**Table 1**). Most micronutrients were detected in serum. Naziroğlu et al. (26) analyzed plasma and erythrocytes; La Serra et al. (23) measured lead in whole blood; the researchers (29) examined coenzyme Q10 and gene expression in skin tissue, respectively, as presented in **Table 1**. All extracted data were cross-checked against the original articles, and any uncertainties were resolved through discussion with the co-authors (X.W., J.D., and W-L.Z.).

### 2.6 Quality assessment

Newcastle-Ottawa Scale (NOS) was utilized to assess the methodological quality of the included studies independently by two authors (H-M.C. and X.W). Any conflicts concerning quality assessment were settled through negotiation with other authors (J.D. and W-L.Z.). The NOS assesses three domains: selection of study groups (maximum 4 points), comparability of study groups (maximum 2 points), and ascertainment of exposure/outcome (maximum 3 points), yielding a total score ranging from 0 to 9. Studies with scores ≥7 were regarded as high-quality; scores of 5-6 indicated moderate quality; scores <5 indicated low-quality (30).

## 3 Results

Based on the literature review, vitamin B3 and vitamin D alleviate the symptoms of pemphigus through immune regulation and anti-inflammatory effects. Meanwhile, antioxidants exert their regulatory role by reducing ROS production and preserving cellular adhesion. Furthermore, micronutrients—including vitamin B3, vitamin D, and antioxidants—may play a pivotal role in the prevention and treatment of pemphigus.

## 4 Discussion - the impact of micronutrients on pemphigus

### 4.1 Vitamins

#### 4.1.1 Vitamin A

Vitamin A, collectively termed retinoids, encompasses a family of lipid-soluble compounds critical for epidermal homeostasis. And its deficiency is well-documented to induce diverse dermatological aberrations (31). Accumulating preclinical and clinical evidence attests to the therapeutic potential of vitamin A and its derivatives in managing oral mucosal disorders, including oral lichen planus (OLP) and pemphigus (32). Pioneering *in vitro* studies by Thivolet et al. demonstrated that retinoic acid (RA), the biologically active form of vitamin A, upregulated the expression of pemphigus IgG-targeted antigens in cultured human keratinocytes while altered the subcellular localization of bullous pemphigoid antigens (32). This finding aligns with the core pathogenic cascade of pemphigus, wherein IgG autoantibodies targeting Dsg1 and Dsg3 disrupt interkeratinocyte adhesion, triggering acantholysis and the classic clinical presentation of flaccid blisters and mucocutaneous erosions (4).

The mechanistic basis of vitamin A’s action in pemphigus hinges on its modulation of keratinocyte function via the classic nuclear receptor pathway. Specifically, RA binds to retinoic acid receptors (RARs) and retinoid X receptors (RXRs) in keratinocytes to form functional heterodimers, which subsequently translocate to the nucleus and bind to retinoic acid response elements (RAREs) in target genes to initiate transcription (31). This signaling cascade directly reinforces epidermal adhesion by upregulating the expression of Dsg1/3 and desmosomal plaque proteins, thereby stabilizing desmosomal structures and counteracting autoantibody-mediated adhesion disruption—ultimately preventing spinous layer loosening (32).

Clinically, the therapeutic utility of retinoids has been validated in recalcitrant pemphigus subtypes. For instance, acitretin (10–30 mg/d) has demonstrated efficacy in treating IgA pemphigus patient’s refractory to conventional steroids and immunosuppressants, achieving partial to complete disease response (33, 34). Alitretinoin, another synthetic retinoid, has also shown promise as a steroid-sparing agent in such cases, with a more favorable side effect profile compared to traditional immunosuppressants (33). These clinical observations corroborate the mechanistic insights, positioning vitamin A derivatives as potential adjuvant therapeutic agents for pemphigus. However, the clinical application of retinoids necessitates careful dose titration and monitoring of adverse effects—including skin dryness, hyperlipidemia, and teratogenicity in reproductive-age females (35).

#### 4.1.2 Vitamin B

Vitamin B complex, including vitamins B3, B4, B6, and B12, represents a category of essential micronutrients that are exclusively synthesized by prokaryotes (36). As critical coenzymes, these vitamins play indispensable roles in the metabolism of amino acids, fatty acids, and glucose, as well as participated in DNA synthesis and methylation processes (37, 38). Clinical evidence has demonstrated that vitamin B deficiency is closely associated with the pathogenesis of various oral mucosal disorders and systemic autoimmune diseases (11).

As the amide form of vitamin B3, nicotinamide serves as a precursor of coenzymes required for a wide array of physiological reactions (39). It exerts anti-inflammatory effects by inhibiting the release of various inflammatory mediators and histamine, consequently reducing leukocyte chemotaxis and lymphocyte transformation (40). Favorable therapeutic outcomes in pemphigus management have been achieved by combining vitamin B3 with antibiotics such as tetracycline or minocycline. The combination of tetracycline (or minocycline) and nicotinamide exerts its curative effects through three core mechanisms: anti-inflammation, immune regulation, and matrix metalloproteinase (MMP) inhibition (40–42). Specifically, tetracycline directly targets the inflammatory cascade in pemphigus by suppressing neutrophil chemotaxis and lysosomal enzyme activity. In addition, nicotinamide modulates immune responses by stabilizing mast cells, inhibiting the production of lymphocyte-derived factors, and blocking antibody-mediated cytotoxicity (40, 41). Finally, the two agents act synergistically to inhibit MMP expression, thereby protecting the extracellular matrix from degradation. The primary advantage of this combination therapy lies in its low toxicity, which renders it a promising alternative or adjunct to corticosteroids. This, in turn, minimizes the adverse effects associated with long-term corticosteroid administration (42).

#### 4.1.3 Vitamin D

Vitamin D is a fat-soluble steroid hormone with dual physiological functions: it not only maintains bone metabolism homeostasis but also serves as a key regulator of the immune system. In recent decades, global vitamin D deficiency has become a prevalent public health issue. An epidemiological study conducted in Pakistan reported that the prevalence of vitamin D deficiency and insufficiency in the general population reached 58.17% and 26.65%, respectively (43). Accumulating evidence has indicated that vitamin D deficiency is closely associated with the incidence and severity of various autoimmune diseases, including pemphigus (44). Clinical observations consistently show reduced serum vitamin D concentrations in patients with pemphigus, suggesting a potential regulatory role of vitamin D in the pathogenesis of this disease (43, 45).

The biological activity of vitamin D relies on a two-step hydroxylation process *in vivo*. Dietary or cutaneously synthesized vitamin D is first catalyzed by 25-hydroxylase in hepatocytes to form 25-hydroxyvitamin D_2_ (25(OH)D_2_)—the main storage form of vitamin D in the body and a clinical indicator of vitamin D status. A case-control study confirmed that the serum level of 25(OH)D_2_ in PV patients was significantly lower than that in healthy controls, and the reduction was positively correlated with the severity of PV lesions (46). Subsequently, 25(OH)D_2_ binds to vitamin D-binding protein (DBP) and is transported to the kidneys and peripheral target tissues (e.g., immune cells, keratinocytes). In these tissues, 1α-hydroxylase further catalyzes 25(OH)D_2_ to generate 1,25-dihydroxyvitamin D_3_ (1,25(OH)_2_D_3_)—the biologically active form of vitamin D (47). Once formed, 1,25(OH)_2_D_3_ enters the nucleus and binds to the VDR, forming a heterodimer complex with the RXR. This complex then binds to specific vitamin D response elements (VDREs) in the promoter regions of target genes, thereby regulating the functions of immune cells and keratinocytes (48).

The pathogenesis of pemphigus is characterized by T cell-driven autoimmune responses, which induce B cells to produce autoantibodies against desmogleins (Dsg) (49). 1,25(OH)_2_D_3_ exerts its immunomodulatory effects primarily through the VDR signaling pathway. On one hand, it promotes the differentiation of regulatory T cells (Treg) by upregulating the expression of forkhead box P3 (Foxp3)—a key transcription factor for Treg cells—thereby inducing the differentiation of naive T cells into functional Treg cells that secrete anti-inflammatory cytokines such as interleukin-10 (IL-10) to suppress excessive immune responses (50). On the other hand, 1,25(OH)_2_D_3_ modulates the polarization of effector T cells (Teff), shifting them from pro-inflammatory T helper 1 (Th1) and Th17 subtypes to anti-inflammatory Th2 subtype by inhibiting the secretion of Th1-related cytokines (e.g., interferon-γ, IFN-γ) and Th17-related cytokines (e.g., IL-17), while promoting the production of Th2-related cytokines (e.g., IL-4) (51). Additionally, 1,25(OH)_2_D_3_ upregulates the expression of indoleamine 2,3-dioxygenase (IDO)—a rate-limiting enzyme in the tryptophan metabolism pathway—in immune cells and keratinocytes. IDO exerts dual immunomodulatory effects: it depletes tryptophan in the local microenvironment, which is essential for the proliferation and activation of Teff cells, thereby inhibiting the function of Th1/Th17 cells, whereas it generates immunomodulatory metabolites (e.g., kynurenine) that directly promote the differentiation and functional maturation of Treg cells. This IDO-mediated pathway synergizes with the Treg polarization effect of 1,25(OH)_2_D_3_ to enhance immune suppression (52).

Mucosal lesions are a typical clinical feature of PV, and a self-limiting immune regulatory loop mediated by vitamin D has been identified in the mucosal microenvironment. Autoantigens (e.g., Dsg3) in the mucosal tissue activate local Teff cells, which then upregulate the expression of 1α-hydroxylase, enabling Teff cells to synthesize 1,25(OH)_2_D_3_ autonomously within the cell, independent of systemic vitamin D metabolism (52, 53). The 1,25(OH)_2_D_3_ secreted by activated Teff cells then binds to VDR in mucosal epithelial cells, inducing high expression of IDO in these cells. IDO in epithelial cells consumes local tryptophan and produces kynurenine, creating an immunosuppressive microenvironment that not only inhibits the further activation of initially activated Teff cells but also promotes the differentiation of Treg cells, ultimately limiting the expansion of autoimmune responses and preventing excessive damage to mucosal tissue (54).

Beyond regulating the immune response, vitamin D also exerts direct targeted effects on the pathogenesis of pemphigus. 1,25(OH)_2_D_3_ directly induces the apoptosis of activated B cells through the mitochondrial pathway—specifically, by upregulating the expression of pro-apoptotic proteins (e.g., Bax) and downregulating anti-apoptotic proteins (e.g., Bcl-2)—thereby reducing the number of autoantibody-secreting B cells in the body (55). Moreover, Dsg3 is a key autoantigen in PV, and its abnormal expression or degradation is closely related to the formation of blisters. Studies have shown that 1,25(OH)_2_D_3_ directly downregulates the mRNA level of Dsg3 at the post-transcriptional level, and this regulatory effect does not require *de novo* protein synthesis, suggesting that 1,25(OH)_2_D_3_ may target the stability of Dsg3 mRNA (56). In addition to direct regulation, sufficient vitamin D indirectly reduces Dsg3 expression by inhibiting B cells from producing anti-Dsg3 IgG antibodies and modulating the composition of T cell subsets (e.g., increasing Treg cells to suppress autoimmunity) (14).

Clinical evidence to date demonstrates that vitamin D supplementation has been reported to improve treatment response in some studies, accelerate corticosteroid tapering, reduce Vitamin D supplementation has been associated with improved treatment response, accelerated corticosteroid tapering, and reduced steroid-related adverse events in some studies, providing a theoretical basis for the potential application of vitamin D supplementation as an adjuvant therapy for pemphigus. Future studies addressing dosage standardization, autoantibody correlation, and drug interactions will further unlock the potential of vitamin D in pemphigus management, ultimately providing a safer and more effective therapeutic option for patients with this chronic autoimmune disease (**Figure 2**).

**Figure 2:**
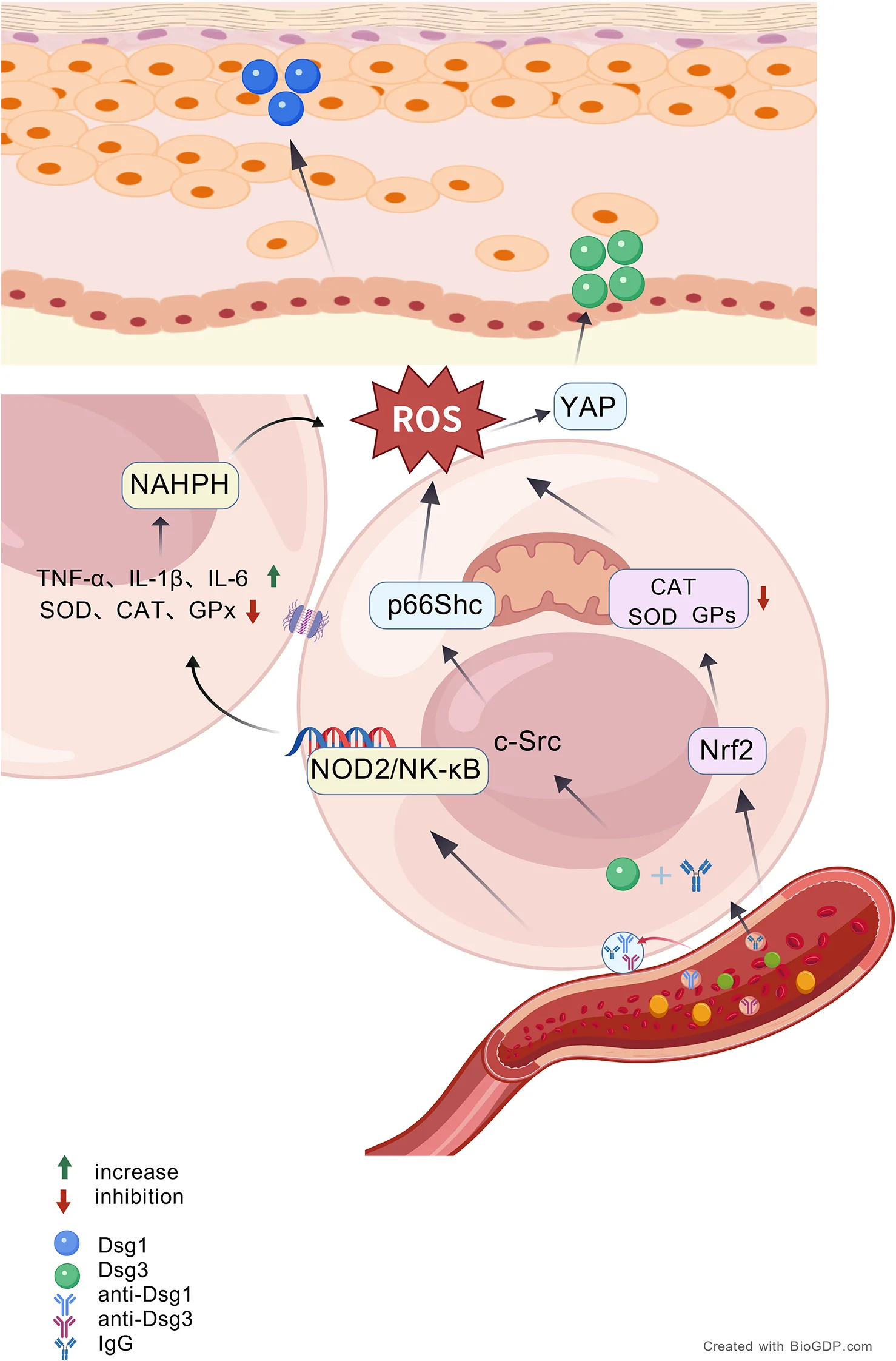
The mechanism of vitamin D in pemphigus. Activated Teff cells synthesize 1,25(OH)_2_D_3_. 1,25(OH)_2_D_3_ forms a heterodimer complex with RXR through VDR, regulating downstream gene expression. This complex promotes Treg differentiation and enhances IL-10 differentiation through Foxp3. 1,25(OH)_2_D_3_ inhibits pro-inflammatory Th1/Th17 cells and promotes the transformation of pro-inflammatory Th1 cells to anti-inflammatory Th2 cells. Mucosal epithelial cells activated by vitamin D signaling upregulate IDO expression. IDO depletes local tryptophan and generates immunoregulatory metabolites, suppressing abnormal immune responses against Dsg3 and potentially indirectly reducing IgG production. 1,25(OH)_2_D_3_ D_3_ enters the nuclei of keratinocytes, binds to VDR, and directly downregulates the gene expression of Dsg3, thereby reducing protein synthesis. These processes collectively alleviate the formation of acantholysis. Figure created using biorender.com.

Activated Teff cells synthesize 1,25(OH)_2_D_3_. 1,25(OH)_2_D_3_ forms a heterodimer complex with RXR through VDR, regulating downstream gene expression. This complex promotes Treg differ-entiation and enhances IL-10 differentiation through Foxp3. 1,25 (OH)_2_D_3_ inhibits pro-inflammatory Th1/Th17 cells and promotes the transformation of pro-inflammatory Th1 cells to anti-inflam-matory Th2 cells. Mucosal epithelial cells activated by vitamin D signaling upregulate IDO expression. IDO depletes local tryptophan and generates immunoregulatory metabolites, suppressing abnor-mal immune responses against Dsg3 and potentially indirectly reducing IgG production. 1,25(OH)_2_D_3_ D_3_ enters the nuclei of keratinocytes, binds to VDR, and directly downregulates the gene expression of Dsg3, thereby reducing protein synthesis. These processes collectively alleviate the formation of acantholysis.

### 4.2 Essential trace elements

#### 4.2.1 Copper

Copper, an essential ultratrace micronutrient, is pivotal for sustaining a broad spectrum of normal physiological functions in the human body (57). Although only trace quantities are required to support core biological processes, excessive systemic accumulation of this metal can elicit pronounced cellular toxicity and a cascade of adverse systemic effects. A key physiological role of copper lies in tissue regeneration and repair: it serves as an indispensable cofactor for lysyl oxidase and other matrix-related enzymes, which mediate the cross-linking of collagen and elastin fibers—the structural cornerstones of the extracellular matrix that maintain tissue integrity (21, 58).

Some clinical evidence suggests an association between copper homeostasis and the pathogenesis of pemphigus. Yazdanpanah et al. reported that serum copper concentrations were significantly lower in 25 pemphigus vulgaris patients compared to 25 healthy controls (*p* < 0.01) (24). To elucidate how copper deficiency contributes to pemphigus, the authors examined its effects on keratinocyte function and immune regulation.

The pathological implications of copper dysregulation in pemphigus are primarily mediated through two interconnected molecular pathways. First, the reduction in serum copper directly impairs the catalytic activity of copper/zinc superoxide dismutase (Cu/Zn-SOD)—a key antioxidant enzyme responsible for scavenging reactive oxygen species (ROS). This impairment disrupts ROS clearance within keratinocytes, and the subsequent accumulation of ROS exacerbates oxidative stress. Elevated oxidative stress, in turn, accelerates the degradation of desmosomal structures—a hallmark event that drives blister formation in pemphigus (59). Second, perturbations in copper metabolism disrupt the balance of T lymphocyte subsets: specifically, it promotes the differentiation of pro-inflammatory Th17 cells while compromising the immunosuppressive function of regulatory T (Treg) cells. This pro-inflammatory shift in the T cell landscape amplifies autoimmune attacks against epidermal keratinocytes, thereby perpetuating disease progression (59).Collectively, these processes form a copper deficiency-driven vicious cycle that amplifies pemphigus disease activity.

However, Javanbakht et al. found no significant difference in serum copper levels between pemphigus patients and healthy controls (*p* > 0.05) (21). In conclusion, the clinical evidence for copper dysregulation in pemphigus remains conflicting, and the biological relevance of the proposed mechanisms requires further investigation.

#### 4.2.2 Iron and selenium

Iron is an essential micronutrient for biological systems, but it also catalyzes free radical generation—with excess iron inducing tissue damage via oxidative stress (60). This effect is potentiated by selenium deficiency, as the two factors act synergistically: iron-driven free radical production is amplified when selenium-dependent antioxidant defenses are impaired. Selenium, a core component of glutathione peroxidase, maintains redox homeostasis by neutralizing ROS (61); its deficiency weakens this protective mechanism, collectively exacerbating oxidative injury in immune-mediated skin disorders (62). Consistent with this, a case-control study of 43 newly diagnosed patients confirmed that serum selenium concentrations are significantly lower in PV patients than in healthy controls (*p* < 0.001) (21), a finding supported by meta-analyses linking selenium depletion to bullous skin diseases (62).

### 4.3 Oxidant and antioxidants

The role of the cellular redox environment in skin homeostasis is widely recognized, as it maintains a dynamic balance between pro-oxidative and antioxidative stimuli—this balance serves as the core foundation for sustaining normal epidermal barrier function and immune homeostasis (25). To preserve this equilibrium, the skin harbors a sophisticated antioxidant system composed of enzymatic components (e.g., superoxide dismutase [SOD], glutathione peroxidase [GPx]) and non-enzymatic mediators (e.g., vitamin E, β-carotene, selenium), which collectively neutralize potential oxidative damage (25).

ROS are endogenously generated during physiological processes such as mitochondrial respiration and phagocytosis, but excessive ROS accumulation triggers detrimental oxidative reactions that disrupt biomolecular integrity (25, 63).The skin, as the primary interface with the external environment, is particularly vulnerable to endogenous (e.g., inflammatory cell activation) and exogenous (e.g., UV radiation) pro-oxidants. Clinically, this vulnerability is evident in autoimmune skin diseases including PV and OLP, where patients exhibit significantly elevated ROS levels and enhanced lipid peroxidation markers (e.g., malondialdehyde [MDA], conjugated dienes) in both lesional skin and systemic circulation (25, 64).

The abnormal elevation of ROS in PV directly reflects disrupted redox homeostasis, driven by synergistic factors: activated neutrophils produce bursts of ROS, while circulating levels of antioxidant vitamins (A, E) and trace elements (copper, zinc, selenium) are reduced—with selenium deficiency correlating negatively with disease duration (26). This imbalance amplifies OS, creating a feedforward loop that exacerbates ROS accumulation and tissue damage (26).

It is well established that autoantibodies targeting Dsg3 and Dsg1 induce acantholysis by impairing desmosome function, with OS acting as a critical mediator across interconnected signaling pathways (65). First, pathogenic antibodies directly compromise antioxidant defenses: the anti-Dsg3 monoclonal antibody AK23 downregulates the transcription factor Nrf2—a master regulator of cytoprotective genes—weakening keratinocyte antioxidant capacity and promoting ROS overproduction (66). Notably, Nrf2 activation is normally triggered by electrophiles that inactivate its inhibitor KEAP1, but this pathway is suppressed in PV, disrupting redox resilience (67).

Second, autoantibodies initiate an inflammatory-oxidative burst via the NOD2/NF-κB pathway. PV patient sera activate NOD2, leading to NF-κB-dependent upregulation of ROS and downregulation of antioxidant enzymes (e.g., SOD, GPx); this OS environment drives proteolytic degradation of Dsg3 and Dsg1, culminating in acantholysis (68). Conversely, naringenin-mediated NOD2 inhibition suppresses NF-κB activation, reduces ROS generation, and preserves Dsg expression to maintain cell adhesion (68). Importantly, Nrf2 and NF-κB pathways act antagonistically in PV, so impaired Nrf2 signaling could enhance NF-κB-driven inflammation (67).

Third, antibody binding triggers a self-amplifying OS-YAP regulatory loop. The binding between PV IgG and Dsg3 upregulates ROS, which downregulates α-catenin (a negative YAP regulator) and activates p38MAPK—these events promote nuclear accumulation and activation of the transcriptional coactivator YAP (68, 69). Activated YAP suppresses Dsg3 gene expression, further weakening intercellular junctions and increasing ROS production, thereby perpetuating acantholysis (69). Antioxidant treatment reverses this loop by reducing YAP accumulation and restoring cell-cell adhesion (69). In addition, synergistic interactions with other pro-oxidative factors exacerbate pathogenesis: oxidized lipoprotein(a)[oxLp(a)] facilitates ROS generation while downregulating Dsg1 expression, further destabilizing desmosomal integrity (70).

Collectively, abnormal ROS elevation and OS activation are key drivers of PV pathological progression. Through a complex network involving Nrf2 inhibition, NF-κB-mediated inflammation, and aberrant YAP signaling, OS disrupts desmosomal function and induces acantholysis—highlighting redox homeostasis as a potential therapeutic target (**Figure 3**).

**Figure 3:**
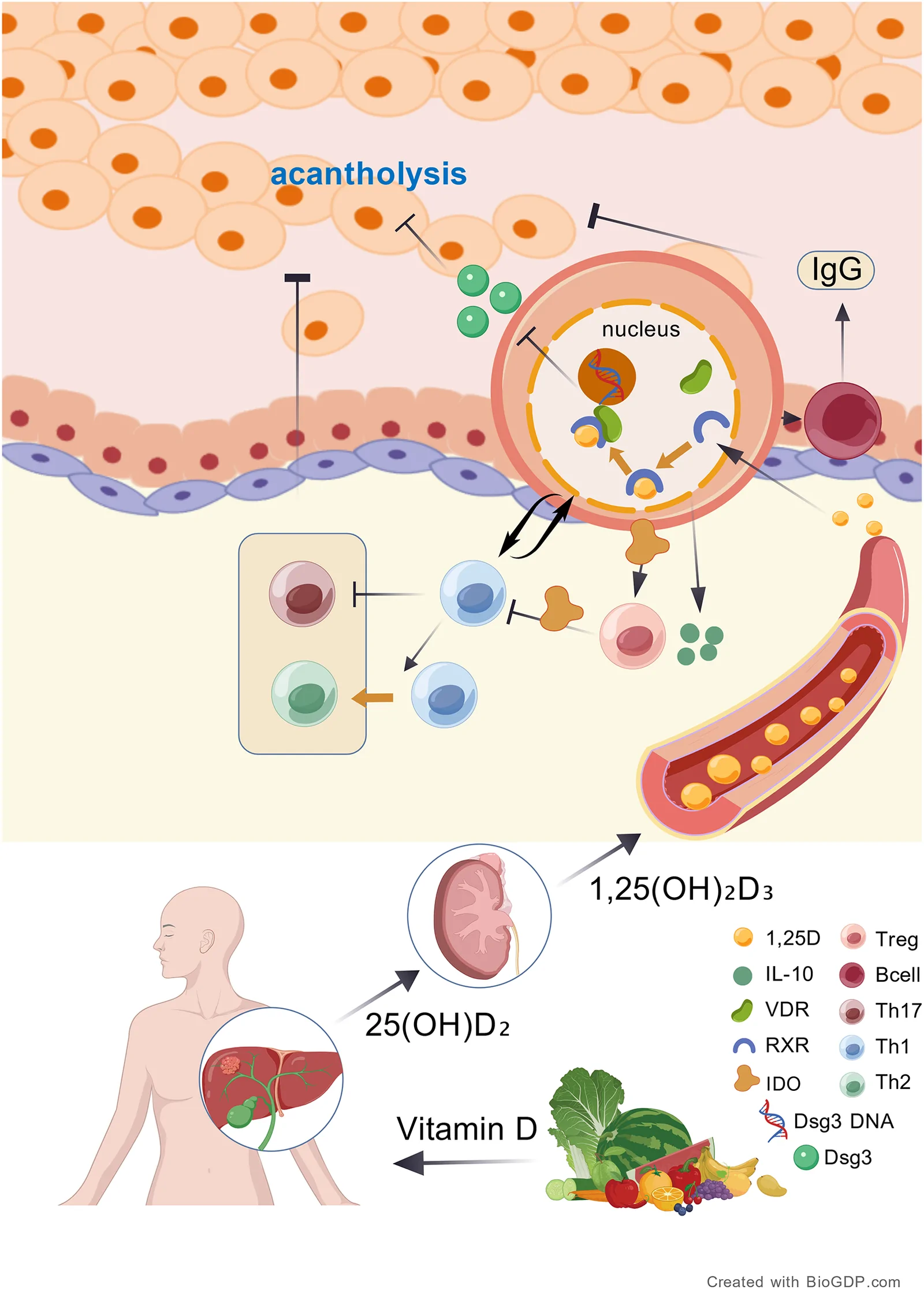
The mechanism of ROS in pemphigus. Figure created using biorender.com.

1. The direct binding between antibodies and Nrf2 decreased the expression of Nrf2 and antioxidant enzymes (SOD, CAT, GPx), thereby weakening the cell’s ability to clear ROS and causing basal ROS accumulation.

2. Antibodies activate the NOD2/NF-κB pathway, and induced the release of large amounts of pro-inflammatory factors such as TNF-α and IL-1β in keratinocytes. These factors further activate NADPH oxidase, producing excessive ROS and exacerbating local inflammation.

3. After antibodies bind to Dsg3, intracellular c-Src kinase is activated, which phosphorylates and activates the mitochondrial protein p66Shc, leading to a burst of mitochondria-derived ROS. High levels of ROS activate p38MAPK and downregulate α-catenin, jointly driving the nuclear translocation of the transcriptional co-activator YAP. YAP, in turn, suppresses the transcription of adhesion proteins, including Dsg3, forming a vicious cycle of “ROS ↑ → Dsg3 ↓ → ROS ↑”.

## 5 Future prospects

In view of the above-mentioned facts, incorporating micronutrients into the daily routine of pemphigus patients may be a promising adjunct therapy to improve the treatment outcomes and alleviate the adverse effects of current treatments. Evidence suggests a positive association between pemphigus and the consumption of certain foods, such as processed meat, sugar-sweetened beverages, and red meat (71). These findings support the potential application of dietary interventions in pemphigus prevention; however, large-scale longitudinal cohort studies and randomized controlled trials are still needed to validate these results.

Additionally, it has been proved that gut microbiome could be the suppliers of certain micronutriets to their host, such as vitamins of B-groups (72). Wang et al. revealed that the gut microbiome and its metabolites are correlated with the onset of PV and the treatment response in PV patients (73). Micronutrients such as vitamin D and antioxidants can modulate the gut microbiota, thereby profoundly influencing the pathological progression of pemphigus. This suggests that targeted dietary supplementation with foods rich in specific micronutrients may become a potential nutritional intervention strategy to support conventional treatment and improve patient outcomes by modulating the microbiota in the future.

## Data Availability

The original contributions presented in the study are included in the article/supplementary material. Further inquiries can be directed to the corresponding authors.
